# Understanding public perception of coronavirus disease 2019 (COVID-19) social distancing on Twitter

**DOI:** 10.1017/ice.2020.406

**Published:** 2020-08-06

**Authors:** Sameh N. Saleh, Christoph U. Lehmann, Samuel A. McDonald, Mujeeb A. Basit, Richard J. Medford

**Affiliations:** 1 Department of Internal Medicine, University of Texas Southwestern Medical Center, Dallas, Texas; 2 Clinical Informatics Center, University of Texas Southwestern Medical Center, Dallas, Texas; 3 Department of Pediatrics, University of Texas Southwestern Medical Center, Dallas, Texas; 4 Department of Emergency Medicine, University of Texas Southwestern Medical Center, Dallas, Texas

## Abstract

**Objective:**

Social distancing policies are key in curtailing severe acute respiratory coronavirus virus 2 (SARS-CoV-2) spread, but their effectiveness is heavily contingent on public understanding and collective adherence. We studied public perception of social distancing through organic, large-scale discussion on Twitter.

**Design:**

Retrospective cross-sectional study.

**Methods:**

Between March 27 and April 10, 2020, we retrieved English-only tweets matching two trending social distancing hashtags, #socialdistancing and #stayathome. We analyzed the tweets using natural language processing and machine-learning models, and we conducted a sentiment analysis to identify emotions and polarity. We evaluated the subjectivity of tweets and estimated the frequency of discussion of social distancing rules. We then identified clusters of discussion using topic modeling and associated sentiments.

**Results:**

We studied a sample of 574,903 tweets. For both hashtags, polarity was positive (mean, 0.148; SD, 0.290); only 15% of tweets had negative polarity. Tweets were more likely to be objective (median, 0.40; IQR, 0–0.6) with ~30% of tweets labeled as completely objective (labeled as 0 in range from 0 to 1). Approximately half of tweets (50.4%) primarily expressed joy and one-fifth expressed fear and surprise. Each correlated well with topic clusters identified by frequency including leisure and community support (ie, joy), concerns about food insecurity and quarantine effects (ie, fear), and unpredictability of coronavirus disease 2019 (COVID-19) and its implications (ie, surprise).

**Conclusions:**

Considering the positive sentiment, preponderance of objective tweets, and topics supporting coping mechanisms, we concluded that Twitter users generally supported social distancing in the early stages of their implementation.

On March 11, 2020, the World Health Organization (WHO) declared the novel coronavirus (COVID-19) outbreak a pandemic and emphasized the need for global governmental commitment to control the threat, citing then 118,319 confirmed cases and 4,292 deaths worldwide.^1^ To contain severe acute respiratory coronavirus virus 2 (SARS-CoV-2), countries closed their international borders.^2^ Despite travel restrictions, global cases continued to increase^3^ requiring enactment of key community mitigation, which garnered significant public attention.^4,5^ These mitigation strategies, named nonpharmaceutical interventions (NPIs), are approaches outside medications, therapies, and vaccines to prevent further spread of SARS-CoV-2 and to reduce the strain on the healthcare system. NPIs fall under 3 main categories: personal, environmental, and community. Personal NPIs refer to behaviors like staying home when sick, coughing or sneezing in a tissue or elbow, wearing a mask, and washing hands with soap and water or using hand sanitizer. Environmental NPIs refer to appropriate surface cleaning of high-throughput areas and commonly used objects. Community NPIs refer to social distancing and closure of areas where large gatherings may occur, such as schools, businesses, parks, and sporting events. Used previously for other viral outbreaks such as influenza,^6^ social distancing or physical distancing refers to increasing the space between individuals and avoidance of larger gatherings in an attempt to reduce viral transmission.^7^ This community NPI has been a main components of effectively fighting the COVID-19 pandemic.^8–10^

Managing and changing public opinion and behavior are vital for social distancing to successfully slow transmission of COVID-19, preserve hospital resources, and prevent exceeding the healthcare system’s capacity.^11^ To affect public opinion, one must first examine and understand it. Social media, specifically its microblogging platform Twitter, serves as an ideal medium to provide this understanding. Twitter has >145 million daily active users^12^ and allows individuals to post, repost, like, and comment on ‘tweets’ of up to 280 characters. Analysis of Twitter has been used previously within the healthcare realm to understand public sentiment and opinion on topics ranging from diabetes,^13^ cancer therapy,^14^ and novel healthcare policies such as the Affordable Care Act.^15^ Within the field of emerging infectious diseases, Twitter analysis has been used to study public opinion and sentiment on measles,^16^ influenza,^17^ and Zika virus outbreaks.^18^

We hypothesized that performing sentiment, emotion, and content analysis of tweets related to social distancing on Twitter during the COVID-19 pandemic could provide valuable insight into the public’s beliefs and opinions on this policy. We further hypothesized that the knowledge gained could prove valuable for public health communication as well as dissemination and refinement of information strategies.

## Methods

### Data collection and processing

From March 27 to April 10, 2020, we extracted daily relevant samples^19^ of English-only tweets related to social distancing and created a 2-week cross-sectional data set of social media activity. We used the rtweet package^20^ to access Twitter’s application programming interface (API) via RStudio version 1.2.1335 (R Foundation for Statistical Computing, Vienna, Austria). The hashtags #socialdistancing and #stayathome, which were the top trending social distancing hashtags at the time of data extraction, were used to identify tweets related to social distancing. We used 15 of the 89 collected tweet metadata variables in our analysis (Table S1 online). We cleaned the tweets by removing characters and words of no or little analytical value and transforming text to its root form. We used Python version 3.6.1 software (Python Software Foundation, Wilmington, DE) for all data processing and analyses. Further details are discussed in Appendix A (online). Institutional review board approval was not required because this study used only publicly available data.

### Sentiment and emotion analysis

We used Python’s TextBlob library^21^ to perform sentiment analysis for all tweets through natural language processing and text analysis to identify and classify emotions (positive, negative, or neutral) and content topics. TextBlob applies the AFINN sentiment lexicon^22^ from a polarity scale of −1 (most negative) to 1 (most positive). We visualized the polarity distribution using bins for strongly negative (−1 to −0.51), negative (−0.5 to −0.01), neutral (0), positive (0.01 to 0.5), and strongly positive (0.51 to 1). We used a recurrent neural network model developed by Colneric and Demsar to label the primary emotion for each tweet based on Ekman’s emotional classification (anger, disgust, fear, joy, sadness, or surprise).^23^ Using χ^2^ testing and Bonferroni correction to adjust for multiple comparisons, we compared the proportion of each sentiment polarity and emotion for each hashtag. We evaluated changes in effect size between hashtags using the absolute difference in percentage points.

### Subjectivity analysis

We used Python’s TextBlob library to perform subjectivity analysis and labeled each tweet from a range of 0 (objective) to 1 (subjective). Objective tweets relay factual information, whereas subjective tweets typically communicate an opinion or belief. For the 2 hashtags #stayathome and #socialdistancing, we visualized sentiment using a histogram of values and compared the median sentiment between hashtags using the Mann-Whitney U test. Through terminology matching, we used key words present in social distancing rules (eg, “stay at least 6 feet [2 meters] from other people” or “avoid large gatherings”) to identify tweets with potentially objective information about these rules (Table S2 online).^7^ We manually reviewed 5% of the resulting tweet subset to verify what percentage of these tweets truly included information about social distancing rules and extrapolated prevalence for the full subset of tweets.

### Topic modeling

To understand the major topics being discussed in our tweet sample, we applied an unsupervised machine-learning algorithm called Latent Dirichlet Allocation (LDA)^24^ using the *gensim* Python library.^25^ LDA is a commonly used topic-modeling approach to identify clusters of documents (in our case, tweets) by a representative set of words. The most highly weighted words in each cluster provide insight into the content of each topic. LDA requires users to input the number of expected topics. To determine the optimal number of topics, we trained multiple LDA models using different numbers of topics ranging from 4 to 50 and computed a topic coherence score (produced by comparing semantic similarity of a topic’s most highly weighted words) for each LDA model. Selecting the LDA model with the highest score, we ultimately chose 10 topics for the final model. An author without access or insight into the topic model initially labeled the topics using the 20 most frequently used terms ordered by weight. All authors then reached consensus on the topic labels. We identified the prevalence of topics by labeling tweets according to their most dominant topic. We identified example tweets whose content pertained >99% to 1 specific topic (Table 2, last column).

## Results

We extracted 1,352,082 tweets during the 2-week period. After removal of repeat and non-English tweets, 574,903 tweets across 347,142 users (range, 1–836 tweets per user; mean, 1.6 tweets per user) were included in the analysis (Table 1). Of those tweets, 98.3% were unique. The hashtag #socialdistancing was included in 264,254 tweets and #stayathome was included in 332,075 tweets; 21,453 tweets contained both hashtags. Twitter for iPhone was the most commonly used platform (31%), followed by Twitter for Android (27.5%). Moreover, <50% of tweets had media (image or video) and more than one-third had a hyperlink. The median user had >3,000 posts and >400 followers at the time of tweeting. Also, 5% of accounts were verified, signified by a blue badge next to a user’s profile name indicating that an account of public interest is authentic.

**Table 1.** Characteristics of Tweets and Twitter Users^a^

Characteristic	All^b^	#socialdistancing^c^	#stayathome^d^
**Twitter source**			
Twitter for iPhone	178,105 (31.0)	79,739 (30.2)	109,808 (33.1)
Twitter for Android	158,071 (27.5)	54,043 (20.5)	104,747 (31.5)
Twitter Web App	108,826 (18.9)	51,482 (19.5)	61,802 (18.6)
Instagram	38,809 (6.8)	24,938 (9.4)	15,147 (4.6)
Hashtags	3 (2–5)	3 (2–5)	3 (2–5)
Has link	207,465 (36.1)	106,428 (40.3)	108,914 (32.8)
Has media	272,005 (47.3)	131,758 (49.8)	151,429 (45.5)
Mentions user	193,392 (33.6)	89,623 (33.9)	109,197 (32.9)
Includes place	52,020 (9.0)	25,333 (9.6)	28,626 (8.6)
Includes geolocation	12,639 (2.2)	8,337 (3.2)	4,677 (1.4)
Is quoted text	51,422 (8.9)	20,797 (7.9)	32,630 (9.8)
Verified twitter account	27,560 (5.0)	13,463 (5.1)	16,015 (4.8)
User followers	437 (98–1,971)	499 (113–2,356)	392 (87–1,714)
User posts to date	3,026 (584–13,086)	3,455 (729–15,280)	2,668 (485–11,378)

a Median (IQR) is presented for numerical variables. N (%) is presented for categorical variables.

b n = 574,903; 564,886 unique.

c n = 264,254 (46.0%).

d n = 332,075 (57.8%).

**Table 2.** Topic Clusters Identified by Topic Modeling^a^

Possible Topic Label	Tweets/Topic	Mean Sentiment	Mean Subjectivity	Words Contributing to Topic Model (in Decreasing Order of Weight)	Representative Tweet
Public opinions and values	88,993	0.07	0.43	see, say, right, good, want, life, even, back, away, someone, many, cant, never, could, practice, give, stop, feel, way, mean	“Another month of #SocialDistancing I already dread looking at the tweets. Since I already feel Millennials are going to be conveniently blamed for this. Everytime something stupid happens, it’s always millennials faults. #ugh”
Media and entertainment	71,589	0.21	0.39	watch, video, new, game, today, fun, join, book, read, play, share, virtual, friend, zoom, week, kid, show, see, check, online	“We had a surprise 25th virtual #zoom #birthday party for my daughter @[tag] this past weekend. Her friends and family sent a 10 sec birthday video message and we created a video birthday card. SO GREAT & made this #SocialDistancing birthday fun, special & memorable [link]”
Quarantine measures and effects	65,229	0.07	0.35	lockdown, pandemic, virus, death, spread, number, test, covidー, country, corona, new, government, say, follow, state, day, india, april, police, report	“As each area reaches the peak in positive #SARSCoV2 cases, the peak deaths follow around 3 to 4 weeks later as the illness sets in and takes its’ toll on the weak. If we #stayathome we do our part to flatten the spread, reduce the illness and reduce the death. #Socialdistancing [link]”
Thank healthcare and reduce spread	64,488	0.21	0.38	thank, together, staysafe, stay_safe, safe, help, maintain, practice, everyone, health, fight, washyourhands, healthy, family, flattenthecurve, follow, pandemic, protect	“A token of appreciation and respect to all colleagues in #publichealth and Medicine especially those at the frontlines fighting #COVID2019 risking their lives for our safety on this #WorldHealthDay2020. Best way of showing gratitude is to #StayAtHome and help contain the pandemic”
Community support and businesses	56,117	0.18	0.39	help, new, way, use, tip, learn, practice, support, connect, team, community, check, online, call, business, share, contact, great, important, find	“Due to COVID-19 #socialdistancing recommendations, our office is temporarily closed. General reception phone line remains open. You can also reach our representatives through the online contact form. Please check our website regularly for further updates: [link]”
Activities	53,947	0.15	0.37	walk, today, day, run, park, exercise, morning, outside, practice, weekend, around, good, house, dog, great, workout, nice, eat, car, coffee	“Another Lush Evenings Walk with Hubby walking off our Roast Beef Dinner … Bitter cold wind but a lovely stroll in the fresh air …. 1 Exercise a day Now chilled with a Cuppa #walking #SocialDistancing #DailyExercise [link]”
Quarantine and isolation	51,725	0.09	0.34	quarantine, day, lockdown, quarantinelife, selfisolation, isolation, week, corona, covidー, new, cat, pandemic, selfquarantine, today, lol, feel, staysafe, month, mom	“Is it just me or did it feel like the month of March lasted a year? Ready to turn the calendar to a new month. #coronavirus #pandemic #ShelterInPlace #SocialDistancing I’m ready to wake up from this nightmare.”
Spring and good sentiments	45,850	0.26	0.41	day, love, beautiful, staysafe, happy, light, today, spring, enjoy, nature, dog, good, hope, photo, smile, walk, april, sunday, lockdown, good_morning	“@[tag] @[tag] Hi and Good morning share-[name], Thanks, I wish you a magic Wednesday filled with happiness, love, joy, peace, laughs and fun. Enjoy every moment with family and friends. #TakeCare #BeSafe #StayAtHome”
Supplies, food, and orders	41,087	0.084	0.34	order, mask, essential, shop, close, store, food, open, practice, delivery, local, state, use, help, wear, line, buy, grocery, park, customer	“@[tag] @[tag] @[tag] @[tag] @[tag] @[tag] @[tag] @[tag] #Stayathome limits the spread of #coronavirus #PoorPeople and low income Seniors at higher risk, stand in long store lines to buy food. #SNAP #WIC #EBT cards can’t be used to shop online. #Help correct this. [link]”
Music and media sharing	35,878	0.21	0.33	coronalockdown_coronavirus, listen, music, click_link, via, artist, check, great, enjoy, dont_forget, enjoy_friend, song, great_artist, listen_rotation, discover_great, great_unsigned, art, dance, draw, sing	“My new single is NOW FOR SALE on iTUNES!! #newmusicalert #newmusic #chillout #2020music #electronic #electronicmusic #spotify #itunes #applemusic #streaming #corona #coronavirus #socialdistancing #producer #unsignedartist [link]”

a Words contributing to the model are shown in decreasing order of weighting. The topics are labeled manually based on these words. The number of tweets primarily with that topic, mean sentiment, mean subjectivity, and sample tweet are also included.

### Word frequency

**Fig. 1.**
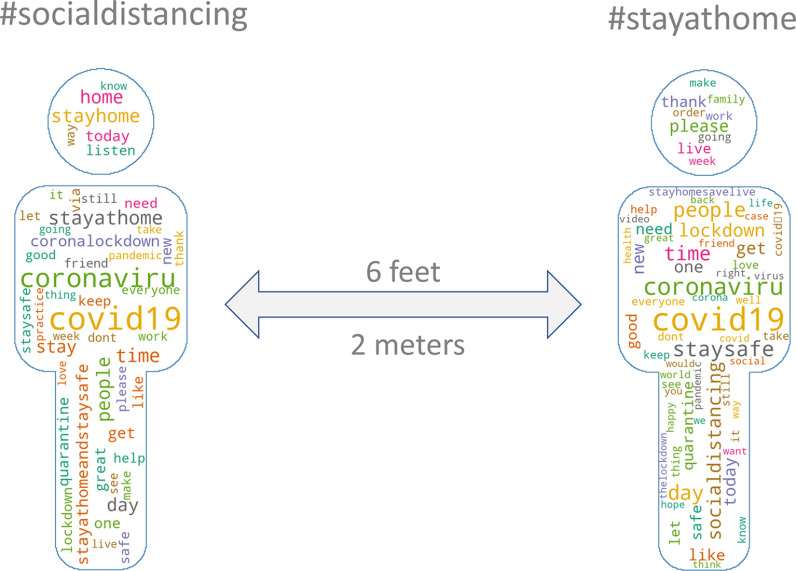
Word cloud of top 200 words.

Our tweet data set contained 13,962,279 words and 93,337,108 characters. The 200 most frequently used words associated with each hashtag before processing are illustrated in Fig. 1. After processing, for both #socialdistancing and #stayathome, the most common word was ‘day’ (20,637 and 28,798 times, respectively). The next 19 most frequent words for #socialdistancing were ‘practice’ (13,988 times), ‘today’ (13,868 times), ‘quarantine’ (13,661 times), ‘coronalockdown coronavirus’ (13,659 times), ‘lockdown’ (12,262 times), ‘help’ (11,064 times), ‘see’ (10,492 times), ‘good’ (10,347 times), ‘new’ (10,204 times), ‘listen’ (9,682 times), ‘staysafe’ (9,609 times), ‘great’ (8,641 times), ‘pandemic’ (8,386 times), ‘way’ (8,243 times), ‘love’ (7,815 times), ‘walk’ (7754 times), ‘say’ (7,167 times), ‘everyone’ (7,141 times), and ‘family’ (6,953 times). For #stayathome the next most frequent words were ‘staysafe’ (21,312 times), ‘lockdown’ (20,839 times), ‘today’ (16,104 times), ‘good’ (13,972 times), ‘quarantine’ (13,758 times), ‘new’ (13,424 times), ‘help’ (13,388 times), ‘see’ (12,545 times), ‘love’ (11,757 times), ‘order’ (10,956 times), ‘everyone’ (9,956 times), ‘say’ (9,836 times), ‘pandemic’ (9,426 times), ‘thank’ (9,280 times), ‘week’ (9,086 times), ‘family’ (9,061 times), ‘life’ (8,793 times), ‘watch’ (8,286 times), and ‘want’ (8,185 times).

### Sentiment polarity analysis

**Fig. 2.**
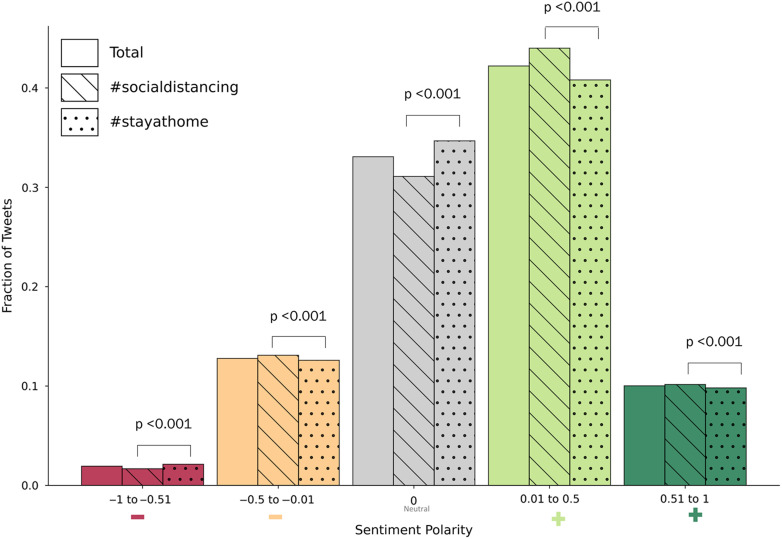
Sentiment analysis for all tweets and stratified by tweets with the hashtag #socialdistancing and #stayathome. Comparison between the two hashtags is done using χ^2^ testing. Bonferroni correction was used to define statistical significance at a threshold of *P* = .01 (0.05/n, where n = 5 since 5 comparisons were completed).

There was net positive sentiment polarity toward both #socialdistancing and #stayathome, with mean polarity scores of 0.150 (standard deviation [SD], 0.292) and 0.144 (SD, 0.287) respectively. Positive and neutral tweets accounting for 52.2% and 33.1% of tweets, respectively (Fig. 2). Moreover, <15% of tweets were negative and <2% were strongly negative. Although statistical differences between polarity categories were detected due to the large sample sizes, the differences in effect sizes were minimal (Fig. 2). Neutral and positive tweets had the largest absolute differences. Compared to #stayathome, #socialdistancing had 3.6% fewer neutral tweets and 3.2% more positive tweets.

### Subjectivity analysis

**Fig. 3.**
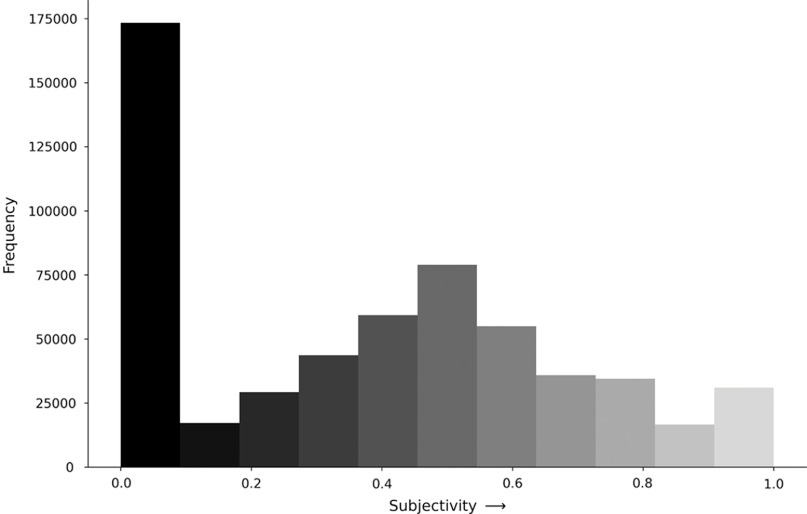
Subjectivity analysis for all tweets. Complete objectivity are defined as 0 and complete subjectivity are defined as 1.

Tweets tended to be more objective in nature and ~30% demonstrated near or complete objectivity (Fig. 3). The median subjectivity scores were similar for #socialdistancing (0.4; interquartile range [IQR], 0–0.59) and #stayathome (0.4; IQR, 0–0.6; *P* = .13). We matched 6,417 tweets that included key words related to social distancing rules and manually reviewed 320 of them. Of the 320 tweets, 249 were confirmed to be related to social distancing rules, yielding a rate of 77.6%. Extrapolating this to all social distancing tweets, we estimate that 4,980 (1.1% of all) tweets referenced social distancing rules.

### Emotion analysis

Joy was the predominant emotion expressed in >50% of tweets with topics ranging from enjoying recreational activities, connecting with family members, and working from home. Examples:

If you are lucky enough to have even a small garden, now is the time to spend sprucing it up. Our spring gardening feature has helpful advice and new ideas to try, to help you make the most of it and #stayathome

and

To bridge the #socialdistancing gap between me & my #grandchildren, I am reading them stories #daily via #zoom & was so pleased to learn about #savewithstories

Fear was the second most common emotion, present in over 20% of tweets. Example:

We need to take the #homequarantine very, very strictly and seriously. if we don’t treat it like a matter of life n [sic] death, it shall definitely become one.. think of it as an army of terrorists outside your door guns.what would you do? !! #stayhome #corona #socialdistancing.

Surprise was the next most prevalent emotion, and tweets included themes of prolonged policy interventions and discovery of novel talents. Examples:

To save lives, #SocialDistancing must continue longer than we expect.

and

I played golf with my wife today. Odd, I didn’t even know she could play. #SocialDistancing, #familytime”

**Fig. 4.**
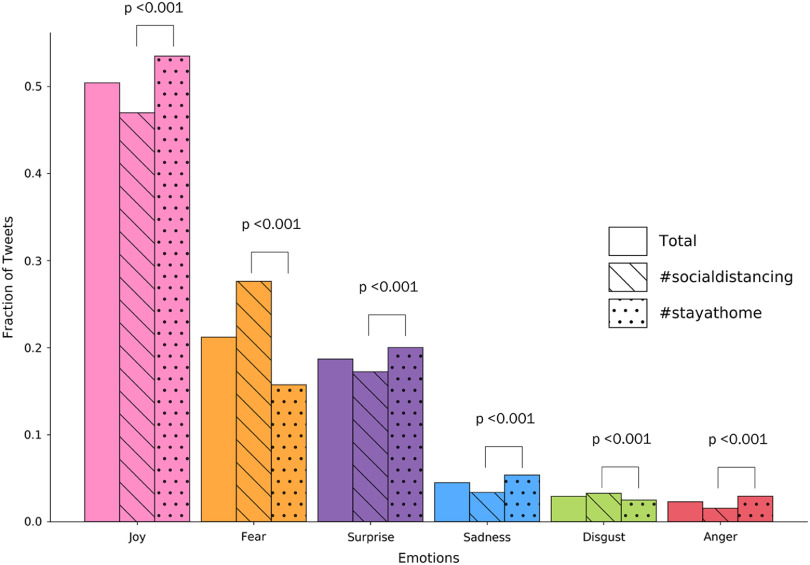
Emotion analysis for all tweets and stratified by tweets with the hashtag #socialdistancing and #stayathome. Comparison between the two hashtags is done using χ^2^ testing. Bonferroni correction was used to define statistical significance at a threshold of *P* = .008 (0.05/n, where n = 6 since 6 comparisons were completed).

The least common emotions found in tweets were sadness, disgust, and anger (Fig. 4). We detected statistical differences in all emotions between #stayathome and #socialdistancing tweets. The largest differences in effect size were joy (#stayathome with 6.6% more) and fear (#socialdistancing with 11.9% more).

### Topic modeling

We identified and subjectively labeled the 10 main tweet topics. Table 2 displays the mean topic sentiment polarity and subjectivity score, key words, and example tweets. “Public opinion and values”, “media and entertainment”, and “quarantine measures and effects” emerged as the three most prevalent topics in 88,993, 71,589, and 65,229 tweets, respectively. Discussion of “spring and good sentiments” had the highest mean polarity of 0.26. “Public opinion and values” and “quarantine measures and effects” had the lowest mean polarity of 0.07. Mean subjectivity scores for all topics ranged from 0.33 to 0.43, with “public opinion and values” having the highest subjectivity score.

## Discussion

Understanding the beliefs, attitudes, and thoughts of individuals and populations can aid public health organizations (eg, the WHO) and government institutions to identify public perception and gaps in communication and knowledge. We analyzed Twitter activity around the 2 most common social distancing trending hashtags at the study time to understand emotions, sentiment polarity, subjectivity, and topics discussed related to this NPI. Tweets predominantly showed positive sentiment polarity. Tweets were primarily linked to emotions of joy (~50%), fear, and surprise. Anger and disgust were the 2 least common emotions expressed. Analyzing key words, we demonstrated that tweets were primarily objective in nature and were used to disseminate public health information. We identified and labeled 10 main topics demonstrating insight into the thoughts and perceptions of the public.

Social media data and channels provide a rich platform to perform public sentiment analysis and have already been used to examine COVID-19 perceptions. One study leveraged social media to distribute a survey to nearly 9,000 individuals in the United States.^26^ Another large study surveyed 6,000 participants in the United Kingdom and the United States.^27^ Despite the robust combined sample size of 15,000 participants, there were inherent limitations to the design. These studies utilize nonprobability sampling like convenience and snowball sampling that are plagued by significant selection bias as well as potential reporting bias, making them prone to sampling error. Through probability sampling from the Twitter API, we analyzed nearly 575,000 English tweets across 350,000 users, providing a broader understanding of public perception that is likely more representational of the population. Using a machine-learning approach, we also explored topics and perceptions without introducing predefined researcher notions, thus limiting the risk of biases inherent to the question design.

Recent public opinion polls from a similar time period have shown that the overwhelming majority of US citizens favored the continuation of social distancing measures.^28,29^ The positive attitude is clearly reflected in the sentiments found in the analyzed tweet sample. Most tweets were either positive or neutral in nature. As public sentiment shifts, we would expect this to be reflected in tweet sentiment as well. For government and public health officials, tweet sentiments may be an important measure to determine the public willingness to continue distancing, which in turn could inform future infection prediction models and social distancing policies.

Many tweets tend to express an opinion; however, tweets associated with #socialdistancing and #stayathome were predominately objective suggesting that these hashtags were used to transmit objective information potentially serving an important public health function. Combined with the large volume of tweets and the finding that 1.1% described social distancing rules, Twitter has the potential to fulfill an important educational function for public health messages.

Joy, fear, and surprise were the dominant emotions for the early phase of social distancing. This correlated well with the topics we discovered, which included leisure activities, community support, and messages of hope (ie, joy), concerns about food insecurity, spreading of the infection, effects of the quarantine (ie, fear), unpredictability of COVID and its unforeseen implications (ie, surprise). As time progresses and the effects of social distancing become more prominent, we would anticipate that other themes such as loss of income, unemployment, inflation, and financial burden would increase in frequency.

The topics we discovered can be aggregated into 4 larger domains. Activities that can be performed during social distancing included 3 topics: media and entertainment, activities, and music and media sharing. Tweets concerning the actual rationale and effect of the social distancing included 3 topics: public opinions and values, quarantine measures and effects, and quarantine and isolation. Two of these were the most prevalent topics. One domain covered the logistics of staying at home falling under a single topic: supplies, food, and orders. The last domain pertained to messages of support and cheering up others: thank healthcare and reduce spread, community support and businesses, spring and good sentiments.

Our study has several limitations. First, we used social media data and specifically Twitter for our analysis. Although there are >300 million monthly active Twitter users, our methodology likely introduced some sampling bias to those with internet and technology access. Second, we used 2 noncomprehensive trending hashtags to identify the most relevant social distancing tweets. We may have missed alternative terminology or key words such as “self isolation” and “corona lockdown,” which appeared as weighted terms in our topic modeling. However, given that these 2 hashtags were the top-trending social distancing hashtags, we expect that these were representative of social distancing during the study period. We recognize that the study period serves as an initial snapshot, rather than a complete evolution, of public perception towards social distancing and that sentiment and topics likely have changed over time. A longitudinal analysis will be a part of future directions. Third, despite analyzing a large number of tweets, we used only a subset of tweets during this time frame, which may have resulted in selection bias. Having analyzed only English tweets, our conclusions may not be generalizable to non-English speaking populations. Since most tweets do not have geolocation, we are also limited in making conclusions based on geographic areas or countries. Fourth, a 2017 study^30^ found that between 9% and 15% of all twitter accounts are bots, which may have affected our analysis. We used the Twitter bot analyzer Botometer^31^ to analyze a random sample of 3,900 users in our dataset. We found that 90% of users have a <20% chance of being a bot. Figure S1 shows the complete probability distribution. Excluding the remaining 10% of users did not change sentiment, emotion, or subjectivity analysis. Finally, we recognize the risk of labeling bias through assignment of topic themes to weighted terms. We attempted to prevent this by having 2 authors perform the topic modeling and 1 author independently perform the labeling task.

In the early phases of social distancing, we were able to successfully obtain and analyze a representative subset of tweets related to this topic. Performing sentiment, emotion, and content analysis of tweets provided valuable insight into the public’s beliefs and opinions on social distancing. Tweets were predominately objective with joy, fear, and surprise as leading emotions. Tweets contained social distancing instructions in >1% of tweets. In the early phases of social distancing, tweets were skewed toward leisure activities and discussion of rationale and effect of social distancing. As social distancing progresses and then is lifted, we anticipate sentiment and topics to change. Although “attitude is only one antecedent of behavior,” the positive emotions, the preponderance of objective tweets, and the topics supporting coping mechanisms led us to conclude that Twitter users generally supported the social distancing measure. Analyzing tweets about nonpharmaceutical interventions such as social distancing based on content, sentiment, and emotion may prove valuable for public health communication, knowledge dissemination, as well as adjustment of mitigation policies in the future. Future research to implement this analysis in real-time using the Twitter Streaming API^32^ could augment directed messaging based on user interest and emotion.

## Supplementary material

For supplementary material accompanying this paper visit http://dx.doi.org/10.1017/ice.2020.406. click here to view supplementary material
