# Supplementary material for: Understanding public perception of coronavirus disease 2019 (COVID-19) social distancing on Twitter
Source: Infect Control Hosp Epidemiol. 2020 Aug 6:1–8. doi: 10.1017/ice.2020.406 (PMC7450231; doi:10.1017/ice.2020.406)
Supplement: Supplementary file 1 [file S0899823X20004067sup001.docx]

**Supplementary Information for:**

Understanding Public Perception of COVID-19 Social Distancing on Twitter

**Authors:**

Sameh N. Saleh, MD^1,2^ [ORCID: 0000-0001-5959-1659]

Christoph U. Lehmann, MD^2^ [ORCID: 0000-0001-9559-4646]

Samuel A. McDonald, MD^2,3^

Mujeeb A. Basit, MD^1,2^ [ORCID: 0000-0002-4948-6158]

Richard J. Medford, MD^1,2^ [ORCID: 0000-0001-9814-8043]

**Corresponding Author:**

Sameh N. Saleh

M: 571-338-3680

F: 214-648-9478

[sameh.n.saleh@gmail.com](mailto:sameh.n.saleh@gmail.com)

**Section A.**

Methods: data processing, transformation, and exploration.

We analyzed tweet metadata to provide descriptive characteristics. For sentiment, emotion, and topic modeling analyses, we transformed tweets into plain text and removed hyperlinks and user mentions. For topic modeling, we further converted the tweet plain text to lowercase, changed words to their root forms (e.g., ‘viruses’ to ‘virus’ or ‘went’ to ‘go’) using the WordNetLemmatizer module of the *NLTK* library in Python(1), and removed stop words (frequently used words with little semantic meaning, such as ‘of’, ‘it’, and ‘is’). For additional dimensionality reduction, we created a list of n-grams (one-word and two-word terms or unigrams and bigrams, respectively) from tweets and removed all extremely low and high frequency terms. These steps decreased the dictionary of terms from 469,039 to 9,984. Using a word cloud, we visualized the top 200 words for each hashtag (after excluding each hashtag and its subdivisions) with larger font size representing greater frequency.

**Table S1.** Metadata associated with tweets

| user_id | lang | retweet_description |
| --- | --- | --- |
| status_id | quoted_status_id | place_url |
| created_at | quoted_text | place_name |
| screen_name  text | quoted_created_at | place_full_name |
| source | quoted_source | place_type |
| display_text_width | quoted_favorite_count | country |
| reply_to_status_id | quoted_retweet_count | country_code |
| reply_to_user_id | quoted_user_id | geo_coords |
| reply_to_screen_name | quoted_screen_name | coords_coords |
| is_quote | quoted_name | bbox_coords |
| is_retweet | quoted_followers_count | status_url |
| favorite_count | quoted_friends_count | name |
| retweet_count | quoted_statuses_count | location |
| quote_count | quoted_location | description |
| reply_count | quoted_description | url |
| hashtags | quoted_verified | protected |
| symbols | retweet_status_id | followers_count |
| urls_url | retweet_text | friends_count |
| urls_t.co | retweet_created_at | listed_count |
| urls_expanded_url | retweet_source | statuses_count |
| media_url | retweet_favorite_count | favourites_count |
| media_t.co | retweet_retweet_count | account_created_at |
| media_expanded_url | retweet_user_id | verified |
| media_type | retweet_screen_name | profile_url |
| ext_media_url | retweet_name | profile_expanded_url |
| ext_media_t.co | retweet_followers_count | account_lang |
| ext_media_expanded_url | retweet_friends_count | profile_banner_url |
| ext_media_type | retweet_statuses_count | profile_image_url |
| mentions_user_id | retweet_location | profile_background_url |
| mentions_screen_name | retweet_verified |  |

**Table S2.** Keywords about social distancing rules

| **Distance** | feet | foot | meter |
| --- | --- | --- | --- |
|  | meters | metre | metres |
| **Crowds** | gathering | crowd | masses |
|  | groups | large groups |  |

**Figure S1.** Probability of user being a bot based on the Botometer tool (2). A random subset of 3,900 users were evaluated.

**References**

1. Loper E, Bird S. NLTK: the Natural Language Toolkit. In: Proceedings of the ACL-02 Workshop on Effective tools and methodologies for teaching natural language processing and computational linguistics - [Internet]. Philadelphia, Pennsylvania: Association for Computational Linguistics; 2002 [cited 2020 Jul 6]. p. 63–70. Available from: http://portal.acm.org/citation.cfm?doid=1118108.1118117

2. Davis CA, Varol O, Ferrara E, Flammini A, Menczer F. BotOrNot: A System to Evaluate Social Bots. In: Proceedings of the 25th International Conference Companion on World Wide Web - WWW ’16 Companion [Internet]. Montr&#233;al, Qu&#233;bec, Canada: ACM Press; 2016 [cited 2020 Jul 18]. p. 273–4. Available from: http://dl.acm.org/citation.cfm?doid=2872518.2889302
